# Delayed recognition of mpox on an inpatient psychiatric unit: a case report and investigation

**DOI:** 10.1017/ash.2025.3

**Published:** 2025-02-11

**Authors:** Waleed Malik, Simon Dosovitz, Jonathan Whitehouse, Nafisa Reza, Sharmin Khan, Justin Chan

**Affiliations:** 1 Division of Infectious Diseases and Immunology, Department of Medicine, NYU School of Medicine, New York, NY, USA; 2 Infection Prevention and Control Department, NYC Health + Hospitals/Bellevue, New York, NY, USA; 3 Department of Psychiatry, NYU School of Medicine, New York, NY, USA; 4 Division of Geriatric Medicine and Palliative Care, Department of Medicine, NYU School of Medicine, New York, NY, USA

## Abstract

This report describes a delayed recognition of mpox in a patient admitted to an inpatient psychiatry unit, resulting in potential exposures to staff and patients. We detail the investigation and risk mitigation efforts and emphasize the importance of prompt identification and isolation in congregate healthcare settings to prevent transmission.

## Background

In 2022, during the global outbreak of mpox clade IIb, New York City (NYC) recorded 3,821 confirmed cases.^1^ Most cases occurred through community transmission, while healthcare-associated transmission was uncommon. However, because mpox had not previously been seen in NYC, healthcare staff may have had difficulty recognizing suspected cases, leading to delays in the implementation of infection prevention and control measures. The risk of transmission from unrecognized mpox is particularly concerning in inpatient psychiatric units, where direct physical contact and exposure to contaminated fomites may be more common in shared spaces. In July 2022, a patient was admitted to an NYC Health + Hospitals/Bellevue (Bellevue) inpatient psychiatry unit with mpox that went unrecognized for 4 days, delaying the implementation of isolation precautions. This report describes the investigation of staff and patients potentially exposed to mpox and outlines lessons learned.

## Case

A man in his 30s with a history of depression and substance use disorder presented to the Bellevue Emergency Department (ED) with suicidal ideation. At ED triage, the patient also reported 2 days of rectal pain, hematochezia, and constipation. He disclosed a history of sexual intercourse with both men and women. Two days prior, he had been presumptively diagnosed as having HSV proctitis at an urgent care clinic but had not taken the prescribed antiviral medication. Physical examination revealed a 0.3 cm scarred, non-tender, non-purulent lesion near the anus and a tender prostate on the rectal exam. Nucleic acid amplification tests for gonorrhea, chlamydia, herpes simplex virus 1 and 2, and varicella-zoster virus, which were negative. He was treated empirically for bacterial prostatitis with doxycycline and ceftriaxone before being admitted to a 28-bed psychiatry unit, where he shared a room with 2 other patients.

On hospital day 2, a computed tomography scan of the abdomen and pelvis revealed no acute pathology. Internal medicine was consulted on day 4 and found 2 tender, ulcerated perianal lesions, along with 2 small scabbing lesions on his trunk and arm. The patient was transferred to a medical unit and placed on airborne and contact isolation for suspected mpox. Swabs of the perianal lesions tested positive for non-variola Orthopoxvirus DNA.

## Investigation

The psychiatry unit where the patient was initially admitted had regular group therapy and mealtimes in common areas, with patients free to interact outside their assigned rooms, though physical contact is prohibited.

Following the patient’s transfer to an isolation room on a medical unit, the psychiatry service collaborated with the hospital epidemiologist to investigate and mitigate any potential healthcare-associated transmission of mpox. Through patient interviews and chart reviews, exposed individuals were categorized according to exposure risk level as defined by the Centers for Disease Control and Prevention (CDC) in July 2022 (Table 1). Exposed patients were restricted from group activities and asked to eat meals in their rooms. Individual therapy and essential medical services were continued. Environmental services performed cleaning and disinfection of all common areas and the index mpox patient’s previous room using bleach-based disinfection products. For 21 days post-exposure, exposed patients were quarantined on the unit, separated from new admissions, and monitored daily for the development of mpox symptoms. Those discharged early were given instructions on community quarantine. For patients who needed transfer to other congregate settings, prior to transfer, we checked if the receiving unit was able to maintain quarantine and symptom monitoring for the remainder of the 21-day period.

**Table 1.** CDC mpox exposure risk criteria

Exposure risk group	Description
**High**	1) Direct contact between a person’s skin or mucous membranes and the skin, lesions, bodily fluids, or contaminated materials from an mpox case 2) Being inside an mpox case’s room or <6 feet during any procedures that may create aerosols from oral secretions, skin lesions, or resuspension of dried exudates (eg, shaking of soiled linens)
**Intermediate**	1) Being within 6 feet for ≥3 h of an unmasked mpox case without wearing, at a minimum, a surgical mask 2) Activities resulting in contact between clothing and the mpox case’s skin lesions or bodily fluids or their soiled linens or dressings
**Low/uncertain**	1) Entered the patient room without wearing eye protection, regardless of duration 2) Being within 6 feet of an unmasked patient for less than 3 h without wearing at minimum, a surgical mask

Note. CDC, Centers for Disease Control and Prevention.These criteria were used in July 2022 by the CDC but have since been updated here: https://www.cdc.gov/mpox/hcp/infection-control/healthcare-settings.html#cdc_generic_section_11-assessing-risk-of-mpxv-exposures-in-healthcare-settings-to-guide-monitoring-and-recommendations-for-postexposure-prophylaxis

Occupational health services assessed all staff members who were on the unit concurrently with the index patient. Staff members were categorized by exposure risk (Table 1), and those exposed were required to report daily temperatures and symptoms for 21 days after the last exposure. None were furloughed from work.

## Results

The investigation identified 84 staff members and 29 patients on unit during the index patient’s stay prior to his isolation (Table 2). Of these, 24 (29%) staff and 29 (100%) patients were considered exposed according to CDC criteria at the time. Exposed staff included physicians, nurses, technicians, social workers, and physical therapists. Eight (15%) exposures were considered high-risk, all among staff. High-risk exposures were due to contact with potentially contaminated linens. All individuals with high-risk exposures were offered the JYNNEOS vaccine as post-exposure prophylaxis, but none accepted.

**Table 2.** Characteristics of persons investigated due to potential exposure to index patient with mpox

Variable	N (%)
**On unit with patient**	**113**
Staff	84 (74)
Patients	29 (26)
**Exposed patients**	**29**
High risk	0 (0)
Intermediate risk	3 (10)
Low risk	19 (90)
**Exposed staff**	**24**
High risk	8 (33)
Intermediate risk	4 (17)
Low risk	12 (50)
**Exposed staff roles**	
Physician	3 (13)
Nursing	12 (50)
Technician	5 (21)
Social work	3 (13)
Physical therapist	1 (4)
**Post-exposure vaccination offered**	**8**
**Vaccination administered**	**0**

By day 21 after the last exposure, no patients or staff had developed symptoms consistent with mpox, and all quarantine precautions were discontinued. By the end of the quarantine period, 6 exposed patients remained admitted at Bellevue.

## Discussion

With mpox still circulating in the United States, healthcare facilities must efficiently identify and isolate suspected cases at points of entry to prevent healthcare-associated transmission. As of July 1, 2024, the Joint Commission regulatory agency delineated new standards that require preparedness for high-consequence infectious diseases at all hospitals.^2^ Mpox presents unique challenges in congregate healthcare settings like inpatient psychiatry units. Its unfamiliarity among clinicians in non-endemic regions, and clinical presentation that can mimic other sexually transmitted infections, can make mpox difficult to recognize.^3^ Moreover, the congregate nature of inpatient psychiatry units can facilitate outbreaks when isolation measures are delayed.^4,5^

Transmission of mpox to healthcare workers due to occupational exposures is rare but well described.^6^ Although most cases of healthcare-associated transmission are due to percutaneous needle stick injuries, there are examples of transmission potentially through contact with contaminated surfaces.^7^ Hospital room surfaces can be contaminated by viable monkeypox virus,^8^ highlighting the importance of environmental disinfection.

Preventing mpox exposure relies on the prompt recognition and isolation of suspected cases at points of entry. However, this can be hampered by busy triage environments, patients unable to provide information due to altered consciousness or limited health literacy, admission of infected patients during the asymptomatic incubation period, or lack of familiarity with infectious diseases by staff trained in nonmedical specialties. In response to this case, we implemented a secondary mpox screening questionnaire to be administered upon arrival to the inpatient psychiatry unit. The goal was to identify suspected cases that may have been missed during the ED course. Since this secondary screening was implemented, we have not experienced any further mpox exposures in our inpatient psychiatry units.

This case also supports the ongoing reassessment of exposure risk criteria based on updated data. In our report, none of those considered exposed based on CDC definitions (Table 1) at the time developed mpox, suggesting those criteria may have overestimated transmission risk. Since then, the CDC’s exposure risk criteria were revised,^9^ potentially reducing unnecessary resource use during exposure investigations. Our experience aligns with previous limited literature indicating an overall low risk of healthcare-associated transmission in well-resourced settings.^10^

This case emphasizes the need for effective point-of-entry screening and prompt isolation of suspected mpox cases. Additionally, the absence of secondary transmission in this investigation helps inform updated exposure risk criteria to ensure resources are focused on managing higher-risk exposures. During outbreaks of unfamiliar pathogens, additional screening measures may be necessary for special populations, such as admissions to congregate healthcare settings, admissions of patients with altered levels of consciousness, or others who may not be able to provide reliable information on initial medical evaluation. Given the difficulty of recognizing emerging infections and the increased risk of exposures in congregate settings, it is vital to enhance healthcare worker education and strengthen screening protocols at points of entry to healthcare settings.
